# Disclosing HIV diagnosis to children in Odi district, South Africa: Reasons for disclosure and non-disclosure

**DOI:** 10.4102/phcfm.v4i1.345

**Published:** 2012-09-07

**Authors:** Johanna M. Mahloko, Sphiwe Madiba

**Affiliations:** 1 Adult Intensive Care Unit, Dr George Mukhari Academic Hospital, Pretoria, South Africa; 2 Department of Environmental and Occupational Health, University of Limpopo (Medunsa Campus), South Africa

## Abstract

**Background:**

The increasing access to antiretroviral therapy (ART) and survival of HIV-infected children has posed challenges to caregivers on disclosing the HIV diagnosis to children.

**Objectives:**

The objectives of this study was to determine the reasons of caregivers for the disclosure and non-disclosure of the HIV diagnosis to children on ART and to determine the caregivers’ perceptions of children's reaction to disclosure.

**Method:**

A cross-sectional study was conducted amongst 149 caregivers of children between 4–17 years who receive ART from a district hospital in South Africa. Descriptive and inferential statistics were used in the analysis of data.

**Results:**

The prevalence of disclosure was 40% and the mean age of disclosure was 9.3 years. Reasons for disclosure included that the child was not adhering to treatment (*n* = 59; 39%); the child was consistently asking questions about the treatment and nature of the disease (*n* = 59; 39%). Reasons for non-disclosure were that the child was too young (*n* = 90; 72%); the child would tell others about diagnosis (*n* = 90; 21.1%); the child would be socially rejected (*n* = 90; 18.6%); fear of negative consequences for the child (*n* = 90; 13.3%); and caregivers do not know how to tell or approach disclosure (*n* = 90; 8.9%).

**Conclusion:**

Caregivers disclosed the diagnosis so that their child would adhere to ART medication; non-disclosing caregivers delayed disclosure because their children were too young to understand the HIV diagnosis. Disclosure of HIV to children should be integrated into regular discussions with caregivers of children in ART settings to improve their knowledge and skills to manage disclosure.

## Abstrait

**Divulguer le diagnostic du VIH aux enfants dans le district d'Odi, Afrique du Sud: Raisons de la divulgation ou de la non-divulgation**

**Contexte:**

Un plus grand accès au traitement antirétroviral (ART) et la survie des enfants infectés par le VIH posent des défis aux soignants sur la divulgation du diagnostic du VIH aux enfants.

**Objectifs:**

Les objectifs de cette étude consistent à déterminer les raisons pour lesquelles les soignants divulguent ou ne divulguent pas un diagnostic du VIH aux enfants sous ART et à déterminer les perceptions des soignants par rapport aux réactions des enfants à la divulgation.

**Méthode:**

Une enquête transversale a été réalisée auprès de 149 soignants d'enfants âgés de 4 à 17 ans qui reçoivent un ART dans un hôpital de district en Afrique du Sud. Des statistiques descriptives et inférentielles ont été utilisées pour l'analyse des données.

**Résultats:**

La prévalence de la divulgation était de 40% et l’âge moyen de la divulgation était de 9.3 ans. Les raisons de la divulgation incluaient le fait que l'enfant ne respectait pas le traitement (*n* = 59; 39%); l'enfant posait constamment des questions sur le traitement et la nature de sa maladie (*n* = 59; 39%). Les raisons de la non-divulgation incluaient le fait que l'enfant était trop jeune (*n* = 90; 72%); l'enfant révèlerait le diagnostic aux autres (*n* = 90; 21.1%); l'enfant serait socialement rejeté (*n* = 90; 18.6%); la peur des conséquences négatives sur l'enfant (*n* = 90; 13.3%); les soignants ne savent pas comment divulguer le diagnostic ou appréhender la divulgation (*n* = 90; 8.9%).

**Conclusion:**

Les soignants divulguent le diagnostic afin que l'enfant respecte la médication de l'ART; les soignants qui refusent de divulguer le diagnostic, reportent la divulgation car l'enfant est trop jeune pour comprendre le diagnostic du VIH. La divulgation du VIH aux enfants devrait être intégrée à un cadre de discussions régulières avec les soignants des enfants sous ART afin d'améliorer leurs connaissances et leurs compétences pour gérer la divulgation.

## Introduction

The Joint United Nations Programme on HIV and AIDS estimates show that 86 270 children in South Africa were on antiretroviral therapy (ART) by the end of 2009.^1^ The increased accessibility of ART in South Africa resulted in more HIV-infected children surviving to older age and adolescence.^2^ As children on ART become older, issues of treatment adherence, self-esteem, bereavement, behavioural problems and fear of illness and death cannot be adequately addressed without disclosure.^3^ In South Africa, few data are available on disclosure of HIV diagnosis of HIV-infected children six years after the commencement of paediatric ART in public health facilities. A recent systematic review of studies conducted on the disclosure of HIV diagnosis to children, revealed only two studies that were conducted in South Africa.^4^ These studies were conducted early in the roll-out of ART in the country.^5, 6^

The increasing survival of HIV infected children has given rise to challenges that caregivers and health care providers face on disclosing the HIV diagnosis to children.^3, 7, 8^ Several studies have documented the benefits of disclosure of the HIV diagnosis to HIV-infected children. Disclosure has been shown by studies from both well developed and developing countries to have positive effects on the clinical course of the disease.^7, 9, 10, 11, 12^ The literature further shows that disclosure is related to good or improved treatment, that enables the children to understand the HIV infection and to make sense of disease-related experiences.^13, 14, 15, 16, 17^ In the context of sex education, disclosure is important for adolescents who may begin to explore their sexuality. It offers a window of opportunity to educate them about preventing further transmission of HIV through sexual activities.^10, 14^

Nevertheless, several studies have documented that many caregivers are reluctant to inform HIV-infected children about their HIV diagnosis.^15, 18, 19^ But very little is known in sub-Saharan Africa about the complex issues of when and how to tell a child about the HIV diagnosis.^20^ However evidence from developing countries shows that caregivers lack knowledge, skills and guidance on how to approach HIV-diagnosis disclosure to children.^5, 12, 21^ Whilst the position of health care providers has been that disclosure of HIV diagnosis to children was a more sensitive and complicated matter than disclosure to adults.^22^ As a result, caregivers struggle with when and how to inform HIV infected children about their diagnosis.^3, 20, 23^

Despite the documented benefits of disclosure, data show that disclosure to HIV-infected children in developing countries is delayed until older childhood and adolescence.^14, 20, 24^ Literature shows that one of the issues that makes disclosure most difficult for caregivers of HIV-positive children is knowing when and how to talk about HIV to these children.^15, 25^ Furthermore, caregivers delay disclosure for fear of negative consequences of disclosure to the child.^12, 26, 27^

### Objectives

The purpose of this study was to determine caregiver's reasons to disclose or not to disclose the HIV diagnosis to HIV-positive children on ART and also to determine caregivers’ perceptions of the child's reaction to the initial disclosure event.

### Contribution to the field

The study will contribute to the limited body of knowledge regarding the decision by caregivers to disclose or not disclose the HIV diagnosis to HIV-infected children. Understanding more about factors that influence disclosure will allow health care professionals providing care to HIV-infected children to better manage the HIV disclosure process.

## Ethical considerations

The Medunsa Research Ethics Committee of the University of Limpopo granted ethical approval for the study. Permission to conduct the study was obtained from the hospital management of Odi hospital and written informed consent was obtained from the caregivers before data collection.

## Methods

### Setting

Data were collected between December 2010 and January 2011. A total of 149 caregivers were recruited from the paediatric HIV clinic in the Odi district hospital in the Bojanala district of the North West Province. The hospital started with adult ART in 2006 whilst the paediatric clinic started providing ART to children from surrounding rural villages and informal settlements during the course of 2010. Most of the children who were receiving ART from this clinic were referred from the tertiary hospital in the district as part of the referral of children to access ART in clinics.

### Design

A cross-sectional study was conducted with caregivers of children on ART using semi- structured questionnaires. Caregivers of children between 4–17 years who were on ART were recruited to participate as they waited for consultation and medication during the routine monthly visits for their children. All caregivers of children aged between 4–17 years who were on ART and who volunteered to participate in the study, were included. For the purpose of this study, we defined a caregiver as the biological mother, biological father, grandmother, grandfather, foster parent, or other relative who performs primary care-giving functions for the child routinely or on a daily basis.

### Procedure

Data were collected by the first author and a trained research assistant. Structured interviews were conducted using a questionnaire developed in English with inputs from literature on disclosure of HIV to children.^28, 29^ The questionnaire was translated into Setswana, the local language of the caregivers in the study area. To ensure quality of the data, the questionnaire was pre-tested and revised prior to the start of data collection. The questionnaire required that caregivers provide socio-demographic information regarding their age, gender, employment status, level of education, marital status, and relationship with the child as well as their HIV status. Caregivers also provided demographic information for the children under their care including age, gender, school grade, diagnosis age, duration on ART, disclosure status, age at disclosure as well as the orphan status of children who were not cared for by biological parents. In addition to structured items, the tool also contained open-ended questions investigating reasons for disclosure and non-disclosure. Caregivers of children who had been disclosed to, were also asked about who disclosed to the child and the child's reactions to disclosure. Caregivers of children who had not been disclosed to were also asked about their intention to disclose.

### Analysis

Data were entered into a Microsoft Excel 2003 spreadsheet and imported to the Stata version 10.0.^30^ First, descriptive statistics were carried out to explore the socio-demographic characteristics of caregivers and children and the results were summarised as frequencies and percentages. The results for numerical variables were summarised as means and standard deviations. *T*-test was used to compare means between children who knew their HIV diagnosis and those who did not know. P-values less than or equal to 0.05 were considered to be statistically significant. Responses to open-ended questions were quantified and also analysed using Stata version 10.0,^30^ and results were summarised as frequencies and percentages.

## Results

### Caregivers’ characteristics

Almost all the caregivers 146 (96.6%) were female, with a mean age of 42.4 years, (s.d. = 14.9, range 19–81 years). The main caregivers were biological mothers 78 (52.3%), followed by grandmothers 42 (28.19%), and 26 (17.4%) were other relatives. Half, 76 (51.0%) of the caregivers had a secondary education, more than half 83 (55.7%) were unemployed, and 21 (14.0%) were pensioners, the majority 64 (42.9%) were single. Almost half, 71 (47.7%) of the children were cared for by people other than their biological parents.

Caregivers were also asked to indicate whether the biological mother of the child in their care was alive; 57 (38.3%) of the children were maternal orphans, whilst the father was not alive in 44 (29.9%) of the cases. More than a third 21 (35.7%) of the children, who were maternal orphans, had lost their fathers too, rendering these children double orphans.

In addition, data were collected on the caregiver's HIV status and more than half 89 (59.7%) of the caregivers were HIV positive, more than a third 55 (36.9%) were HIV negative and only 5 (3.36%) did not know their HIV status. All 78 (100%) of the biological mothers were HIV positive and about 6 (*n* = 36) grandmothers were HIV positive. The caregivers’ characteristics of disclosed and not disclosed children (Table 1).

**TABLE 1:** Caregiver demographic characteristics by disclosure status of children.

Variables	No		Yes		*p*-value
			
	*n*†	%	*n*‡	%	
**Marital status**					0.582
Single	43	47.7	21	35.5	
Married	21	23.3	15	25.4	
Live with partner	11	12.2	10	16.9	
Divorced	3	3.3	4	6.7	
Widowed	12	13.3	9	15.2	
**Employment status**					0.404
Employed	28	31.1	13	22	
Unemployed	49	54.4	34	57.6	
Pensioner	10	11.1	11	18.6	
Schooling	3	3	1	1.6	
**Highest level of education**					0.724
Primary	20	22.2	17	28.8	
Secondary	46	51.1	30	50.8	
Grade 12	21	23.3	10	16.9	
Tertiary	3	3.3	2	3.3	
**Relationship to child**					0.293
Mother	52	57.8	26	44	
Father	2	2.2	1	1.6	
Grandmother	24	26.7	18	30.5	
Other	12	13.3	14	23.7	
**Caregiver HIV status**					0.172
Negative	29	32.2	26	44.1	
Positive	59	65.6	30	50.8	
Unknown	2	2.2	3	5.1	
**Mother alive§**					0.372
No	32	84.2	25	75.8	
Yes	6	15.8	8	24.2	
**Father alive**¶					0.004
No	19	21.6	25	42.4	
Yes	60	68.2	24	40.7	
Unknown	9	10.2	10	16.9	
**Relationship to child**					0.293
Mother	52	57.8	26	44.1	
Father	2	2.2	1	1.7	
Grandmother	24	26.7	18	30.5	
Other	12	13.3	14	23.7	

† *n* = 90.

‡ *n* = 59.

§ *n* = 71.

¶ *n* = 147.

**TABLE 2:** Child characteristics in disclosed and non disclosed group.

Variables	no		yes		*p-*value
			
	*n*†	%	*n*‡	%	
**Age**					< 0.01
4 yrs	31	91.1	3	5	
6–10 yrs	54	67.5	26	44	
11–17 yrs	5	14.2	30	50.8	
**Diagnosis age**					< 0.01
1–5 yrs	67	74.4	23	38.9	
6–10 yrs	23	56	18	30.5	
11–17 yrs	0	0	18	30.5	
**Disclosed age**					< 0.01
4–5 yrs	31	91.1	3	5	
6–10 yrs	54	67.5	26	44	
11–17 yrs	5	14.2	30	50.8	
**Age when ART was initiated**					< 0.01
1–5 yrs	61	67.7	15	25.4	
6–10 yrs	29	32.2	24	40.6	
11–17 yrs	0		20	33.9	
**Duration on ART**					0.43
1–5 yrs	83	92.2	55	93.2	
6–10 yrs	7	7.7	3	5	
11 yrs	0		1	1.6	
**Schooling**					0.002
No	19	90.4	2	9.5	
Yes	71	55.4	57	44.5	

† *n* = 90.

‡ *n* = 59.

### Characteristics of children

A total of 149 caregivers provided information about the children under their care. There were slightly more girls 86 (57.7%) than boys 63 (42.2%) in the sample. The mean age of the children was 8.2 years (s.d. = 3.1, range 4–17 years). All the caregivers provided information on the diagnosis age of the children, the majority 90 (60.39%) were diagnosed between 1 and 5 years, the mean diagnosis age was 5.3 years (s.d. = 3.6). All (100%) of the children were on ART, the mean time on ART was 3.0 years (s.d. = 1.79), and the mean age when children started ART was 5.9 years (s.d. = 3.5). The majority of the children 149 (90.81) were attending school.

### Prevalence of HIV disclosure

The caregivers were asked to indicate whether their children knew their HIV diagnosis, and about 59 (39.6%) of children were told their positive HIV status. The caregivers were asked the age at which the children were informed of their HIV status, more than half 30 (50.85%) learned about their HIV status between 11 and 17 years, 26 (44.06%) between 6 and 10 years and only 3 (5.08%) children were younger than 6 years when they learned about their HIV status. The mean age of disclosure was 9.3 years (s.d. = 2.9, range 4–17 years). The child characteristics of the disclosed and not disclosed groups (Table 1).

Caregivers were asked about the person who informed the children of their HIV diagnosis, and for the majority of children, disclosure was done by their biological mothers 23 (38.98%); almost a third 17 (28.8%) by grandparents; about a quarter 13 (22.03%) by health care providers (health providers were doctors, nurses and social workers); and almost a tenth 5 (8.47%) by other relatives. The 'other relatives’ were older siblings, aunts, and uncles who played the role of informal foster parents.

### Caregivers’ reasons for disclosure

Using open-ended questions, caregivers (*n* = 59) who disclosed the HIV status to the children were asked their reasons for informing their child about the HIV diagnosis. The most common response was that the child was not adhering to treatment or that they disclosed the status so that the child would adhere to treatment 23 (39.0%). Other reasons for disclosure were that the child was consistently asking questions about the reasons why they were taking medication 15 (25.4%) as well as wanting to know the nature of their disease 8 (13.6%). Caregivers also disclosed for fear that the children would learn the HIV diagnosis from other sources 6 (9.5%). For about a tenth 5 (8.5%) of the children, disclosure was prompted by the health care providers. Reasons for disclosure are presented (Table 3).

**TABLE 3:** Reported reasons for non-disclosure amongst caregivers of children on ART (*n* = 59).

Reason	*n*	%
Child is too young to understand	65	72.2
Child will tell others	19	21.1
Child will be stressed and/or hurt and/or worry	12	13.3
Child will be socially rejected/mocked	11	12.2
Caregiver not ready and/or don't know how to tell	8	8.9
Child will ask questions about transmission	2	2.2
Child will be angry with caregiver for being sick	2	2.2
Child not staying with primary caregiver	2	2.2
Child is deaf, caregiver cannot communicate	1	1.1
Child is recently diagnosed	1	1.1

Some caregivers gave more than one reason for non-disclosure; therefore the numbers reported are more than the total (*n* = 59).

### Caregivers’ reasons for non-disclosure

Caregivers who had not informed their children about their HIV diagnosis (*n* = 90) also responded to an open-ended question about their reasons for not disclosing. More than two thirds 65 (72.2%) of the caregivers believed that their child was too young to understand the HIV diagnosis; 19 (21.1%) delayed disclosure for fear that the child would tell others about their diagnosis; 11(18.6%) believed that if the child told others about their diagnosis, they would be socially rejected; 12 (13.3%) delayed disclosure for fear of negative consequences for the child; caregivers believed that disclosure would stress, hurt and worry the child. Lastly caregivers delayed disclosure because they believed that they did not know how to tell or how to approach the disclosure of the HIV diagnosis to their children. The common reasons cited by caregivers for not disclosing the HIV diagnosis to their children (Table 4).

**TABLE 4:** Reported reasons for disclosure amongst caregivers of children of ART (*n* = 90).

Reasons	*n*	%
Child can adhere to medication	23	39
Child was asking questions why he was taking medication	15	25.4
Afraid that child will be socially rejected	11	18.6
Afraid child will learn about HIV diagnosis from other sources	9	15.3
Child wanted to know what he was suffering from	8	13.6
Health care provider told caregiver it was time to tell the child	5	8.5
Child was reaching puberty and/or right age	3	5.1
So child understands the disease/clinic visits/medication	8	13.6
So child can take better care of self	2	3.4

Some caregivers gave more than one reason for disclosure, therefore the numbers reported are more than the total (*n* = 90)

**TABLE 5:** Children's reaction to disclosure (*n* = 59).

Reason	*n*	%
Child showed no signs of negative reaction	36	61.0
Child showed signs of sadness and/or withdrawn	21	35.6
Child was worried and/or depressed and/or tearful	11	18.6
Child showed signs of frustration	6	10.2
Child was surprised	1	1.7
Child showed signs of anger	2	3.4

Some caregivers gave more than one reaction to disclosure therefore the numbers reported are more than the total (*n* = 59)

### Child's emotional response to disclosure

Caregivers (*n* = 59) who had disclosed the HIV diagnosis were further asked in an open-ended question to report on the reactions of the child to the initial HIV-disclosure event. Two-thirds 36 (61.0%) of the caregivers reported that the children showed no reaction following disclosure; a third 21 (35.6%) reported that the children were sad and withdrawn; less than a quarter 11 (10.2%) of caregivers reported that the children were depressed, worried and tearful. The children's common emotional responses to disclosure.

## Discussion

The prevalence of disclosure amongst caregivers of children on ART in our study was 40%, higher than the reported prevalence of 31% in Zambia,^31^ 30.1% in Thailand,^12^ 29% in Uganda,^13^ 21% in Ghana^7^ and 17.4% in Ethiopia.^18^ The higher prevalence rate observed in our study might be attributed to the increased number of older children (mean age of 8.2, range 4–17 years) enrolled for ART in our study setting. Biological mothers were the main caregivers in the sample and made up half of the caregivers, they also disclosed to the majority of children (39%). However, more than half (61%) of the children were actually disclosed to by people other than their biological parents despite the biological mothers being the majority of caregivers. Almost a third of the children were disclosed the HIV diagnosis by the grandparents and about a quarter of the children by the health care providers.

Caregivers in this study disclosed so that the children should adhere to medication. This agrees with findings of studies from developing and developed countries where improving treatment adherence was identified as the most common reason for disclosure.^4, 12, 27, 32, 33^ Disclosure has been reported to positively influences adherence to ART for some HIV-positive children.^18^ In an Ugandan study, disclosure of the HIV diagnosis was necessary to overcome the children's opposition to taking HIV treatment and complete disclosure of the HIV diagnosis to children was related to good ART adherence.^13^ Likewise Vaz et al.,^27^ reported that study caregivers and youths believed that their adherence to treatment improved as a result of being told their HIV diagnosis.

Another reason for disclosure was that the children were persistent in questioning the reasons as to why they were taking medication and also that they wanted to know about the nature of their illness. Our findings are in line with current data from developing countries, showing that disclosure was influenced by the children's questioning and wanting to know about their health.^32^ Similar findings were reported in well-developed countries, caregivers disclosed because of persistent questioning by the children, particularly when adherence to treatment was a problem.^5, 17, 34, 35, 36^ Data showed that when children had been on ART for some time, they no longer experienced symptoms evident in the illness and therefore did not understand why they should continue taking medication.^20^ It is for this reason that disclosure of HIV to children on ART has become an important part of their comprehensive medical care. As already stated, disclosure enables children to understand HIV infection and to make sense of their disease-related experiences and the importance of adherence to the treatment.^7, 14, 17^

Similar to findings from previous studies,^4, 8, 26, 37^ caregivers in this study disclosed the HIV diagnosis because they were concerned that children would learn the HIV diagnosis from other sources. The caregivers believed that learning the diagnosis from other sources would hurt the child and destroy the trust between the caregiver and the child. The caregivers wanted to take a leading role in the disclosure of the HIV diagnosis to their children, as they believed that disclosure should take into consideration the ability of the child to understand HIV-related information and they were in the best position to do that.^38^ In this study, television was one of the potential sources of accidental or unintended disclosure; caregivers were concerned that children would recognise their medication from HIV educational programmes shown on television. This suggests that caregivers who had not disclosed the HIV diagnosis to the children and children in their care were not benefiting from HIV educational programmes aired on the television. Under these circumstances, caregivers who did not disclose the diagnosis were less likely to watch these programmes for fear of unintended disclosure of the HIV diagnosis of their children.

Previous studies conducted in South Africa documented low levels of involvement of health care providers in disclosure of the HIV diagnosis to infected children.^5, 6, 39^ Our findings are similar to recent reports,^20^ which show that health care providers play a large role in initiating disclosure with children. Caregivers in this study reported that disclosure of the HIV diagnosis to their children was prompted by health care providers who often suggested to caregivers to tell the child because it was the right time. The increased involvement of health care providers in HIV disclosure to children observed in this study further demonstrates that discussions about HIV disclosure with HIV-infected children is increasingly becoming part of their comprehensive care in South Africa.

Similar to previous studies,^3, 5, 8, 12, 16, 17, 35, 40^ caregivers in this study delayed disclosure because they believed that the child was too young to understand the HIV diagnosis. According to Lester et al.,^35^ parental worry about the capacity of younger children to understand the HIV diagnosis was often linked to the fear that children would impulsively disclose it to others. Although there is a general lack of consensus about the age at which the child should be told about the HIV diagnosis, caregivers view children below the age of five years as being too young for disclosure.^4, 7, 15, 20, 41, 42^ Our findings support this view; the mean age of disclosure in this study was 9.3 years. With increased access to ART for children soon after diagnosis, the age profile of children on ART is rapidly changing. Health care providers who work with caregivers of HIV-infected children, should be aware of the changing age profile of children when planning for disclosure counselling of caregivers.

Disclosure was also delayed for fear that children would tell others about their diagnosis. Embedded in this concern is the fear of the stigma and discrimination surrounding a positive HIV-status disclosure. Caregivers were concerned that once children told others about their HIV diagnosis they would be socially rejected, mocked, teased, and isolated by their friends at school and in the community. Similar findings on fear of the social stigma were previously reported.^5, 7, 12, 17, 18, 19, 43^ HIV-infected mothers were especially worried about disclosing the HIV diagnosis to their children because of the stigma associated with the HIV diagnosis. Concerns that the child would impulsively tell others about their HIV diagnosis epitomize the secret nature of an HIV diagnosis. Caregivers do not trust the ability of children to keep their diagnosis secret whilst still young.^7, 12, 14^

In addition, caregivers delayed disclosure for fear of negative emotional consequences for the children. Fears that children would be worried, sad, cry, harm themselves, run away, give up all hope or simply die from grief following disclosure, were previously documented as common reasons for non-disclosure.^12, 23, 26, 27^ There is however evidence that feelings of anger, frustration, sadness, worrying, and crying that normally occur after disclosure become less common over time.^14^ It is worthy of note that caregivers who informed children about their HIV diagnosis in this study reported that the majority of the children (61%) did not show any negative reaction following disclosure. However, there is currently inadequate data to explain the neutral response from children following disclosure. It is argued that because many children are often disclosed to at a young age, they may not fully understand the diagnosis.^44^

We also found that caregivers delayed disclosure to children because they believed that they did not know how to tell or how to approach the disclosure process. Similar findings were reported in other studies from developing countries.^5, 12, 21^ As a result, caregivers in this study and other studies,^5, 6, 12, 14, 20, 28, 39^ relied on the support of the health care providers to disclose the HIV diagnosis to children. Added to the lack of disclosure skills, caregivers were also afraid to answer questions about the transmission or sources of the HIV infection.^4, 5, 8, 12, 14, 26, 28, 37, 45, 46, 47, 48^

### Practical implications

There is a need that disclosure of the HIV diagnosis to infected children be integrated into regular discussions with caregivers of children in ART settings. This will facilitate disclosure as both the health care providers and caregivers will be involved in the disclosure of the HIV diagnosis to children.

### Limitations

One of the limitations of this study is that the experiences of the HIV diagnosis disclosure are from the caregiver's perspective, but the voices of the HIV positive child remain unheard. More research is needed to explore disclosure with children, and South Africa is currently in a position to do so given the increased number of children on ART and the improvements observed on the rates of disclosure. Another limitation is recall bias. Caregivers might not recall the children's reaction to disclosure and might have under or over-estimated the reactions. In addition, the caregivers might not recall some of the events in the child's life, such as the age at which the child started with ART and the age at which the child was diagnosed with HIV.

### Recommendations

It is recommended that more research be conducted to understand the factors that influence disclosure to children in order to develop disclosure guidelines that would better support caregivers to disclose the HIV diagnosis to their children. Improving caregiver knowledge and skills to manage the HIV disclosure to their children is critical in increasing the rates of disclosure in developing countries.

## Conclusion

We found that the majority of caregivers disclosed the HIV diagnosis so that their child would adhere to ART medication. The importance placed on the improvement of treatment adherence when caregivers disclosed the HIV diagnosis to children, suggests poor adherence to treatment amongst children who were not informed about the HIV diagnosis. Caregivers who did not disclose the HIV diagnosis, delayed disclosure because their child was too young to understand the HIV diagnosis and would tell others about their diagnosis subjecting the child and the family to social rejection. The fear of negative emotional consequences for children was also a reason for delaying disclosure. However, caregivers who had disclosed reported that the majority of the children did not show any negative reactions following disclosure. Disclosure was also delayed because caregivers believed that they lacked disclosure skills and relied on the support of the health care providers to disclose the HIV status to their children.
